# Probabilistic identification of bacterial essential genes via insertion density using TraDIS data with Tn5 libraries

**DOI:** 10.1093/bioinformatics/btab508

**Published:** 2021-07-13

**Authors:** Valentine U Nlebedim, Roy R Chaudhuri, Kevin Walters

**Affiliations:** Department of Statistics, School of Mathematics, University of Leeds, LS2 9JT, UK; Department of Molecular Biology and Biotechnology, University of Sheffield, Sheffield S10 2TN, UK; School of Mathematics and Statistics, University of Sheffield, Sheffield, S10 2TN, UK

## Abstract

**Motivation:**

Probabilistic Identification of bacterial essential genes using transposon-directed insertion-site sequencing (TraDIS) data based on Tn5 libraries has received relatively little attention in the literature; most methods are designed for mariner transposon insertions. Analysis of Tn5 transposon-based genomic data is challenging due to the high insertion density and genomic resolution. We present a novel probabilistic Bayesian approach for classifying bacterial essential genes using transposon insertion density derived from transposon insertion sequencing data. We implement a Markov chain Monte Carlo sampling procedure to estimate the posterior probability that any given gene is essential. We implement a Bayesian decision theory approach to selecting essential genes. We assess the effectiveness of our approach via analysis of both simulated data and three previously published *Escherichia coli*, *Salmonella* Typhimurium and *Staphylococcus aureus* datasets. These three bacteria have relatively well characterized essential genes which allows us to test our classification procedure using receiver operating characteristic curves and area under the curves. We compare the classification performance with that of Bio-Tradis, a standard tool for bacterial gene classification.

**Results:**

Our method is able to classify genes in the three datasets with areas under the curves between 0.967 and 0.983. Our simulated synthetic datasets show that both the number of insertions and the extent to which insertions are tolerated in the distal regions of essential genes are both important in determining classification accuracy. Importantly our method gives the user the option of classifying essential genes based on the user-supplied costs of false discovery and false non-discovery.

**Availability and implementation:**

An R package that implements the method presented in this paper is available for download from https://github.com/Kevin-walters/insdens.

**Supplementary information:**

Supplementary data are available at *Bioinformatics* online.

## 1 Introduction

Bacterial essential genes are those required for growth and survival (i.e. viability). They are usually defined operationally in natural or specific conditions (Karash and Kwon, 2018) with those genes required for basic metabolism or growth in the natural host or environment identified as essential (Tateishi *et al.*, 2020). Genes required for specific biological processes like motility, drug resistance, cell division etc. can also be identified under specific conditions (Mekalanos *et al.*, 2001). The genes that are required under almost all growth conditions are known to be generally or unconditionally essential. Such genes perform essential functions that include fundamental processes like the DNA replication required in all organisms, as well as other essential functions required for the organism’s particular lifestyle (Chao *et al.*, 2016). Genes that are only required for growth under some specific conditions are referred to as condition-specific essential genes or simply said to be conditionally essential. The conditionally essential genes depend on factors ranging from environmental to genetic context, and the adaptability of the organism to survive inactivation of unconditional essential genes (Chao *et al.*, 2016). Due to the implications for identifying effective and narrower biochemical drug targets, essential genes are of great interest (Kinnings *et al.*, 2010). The ever-growing concern about antibiotic resistance has coincided with revolutionary progress in the availability of genome sequences and high throughput methods to study bacteria (Su *et al.*, 2019). The development of new technologies and approaches has transformed pathogen studies. The development of genome-wide experimental approaches to identify essential bacterial or virulence genes for *in vivo* survival has seen considerable progress, which could yield potential drug targets (Friedman and Hughes, 2003).

A transposon is a sequence of bacterial DNA that can move, either by a ‘copy and paste’ (replication) or ‘cut and paste’ (relocation) mechanism, within or between DNA molecules. Some transposons have specific target sites (like the mariner family of transposons), others [e.g. transposon 5 (Tn5)] insert into almost any target sequence (Hickman and Dyda, 2016). Transposons have implications for the function of essential genes since their insertion into a region of the genome leads to disruption of the processes associated with that region (Judson and Mekalanos, 2000). Essential genes are known to be conservative and usually do not tolerate transposon insertions, except at the distal parts (near the 5’ or 3’ end) (Bourque *et al.*, 2018). This characteristic of essential genes is key to their identification. Recent approaches to the identification of essential genes are based on the development of hybrid methods that integrate transposon mutagenesis with high-throughput sequencing (Chao *et al.*, 2016).

In recent years Transposon Insertion Sequencing (TIS) technologies have been developed. The processes involved in the TIS technique include the construction of large libraries of mutants in which genes are disrupted randomly by transposon insertions. The expectation at this stage is that the created libraries will have significant numbers of insertions only in genes not required for growth. Furthermore, these libraries are grown under certain experimental conditions of interest. Organisms that grow and survive under the specified conditions are those whose disrupted genes are unnecessary for such functions. The use of next-generation sequencing to facilitate high-throughput identification of essential genes compliments experiments using random mutant libraries (Peng *et al.*, 2017). Different applications of second generation sequencing to transposon mutagenesis screens have evolved independently (Chao *et al.*, 2016). Among them are: High-throughput Insertion tracking by deep sequencing (Gawronski *et al.*, 2009), Transposon-directed Insertion-site Sequencing (TraDIS) (Langridge *et al.*, 2009), Insertion Sequencing (INSeq) (Goodman *et al.*, 2009) and Transposon Sequencing (Tn-Seq) (Van Opijnen *et al.*, 2009; Van Opijnen and Camilli, 2013). As highlighted in Chao *et al.* (2016) these TIS approaches are conceptually identical but there are significant differences in the protocols, as reviewed by Van Opijnen and Camilli (2013).

Most of the statistical methods developed to analyse TIS data utilize insertion-level approaches. They rely on information derived from the number of potential insertion sites to identify essential genes in saturated libraries. They are predominantly designed for mariner-based transposons and exploit the preference for TA site insertions in their approaches to classification. The mariner-based transposons enable densely saturated libraries, that have insertions at all/nearly all TA sites, to be constructed. A major advantage of the mariner-based transposons is that insertions sites (TA-sites) are defined and as such has the assumption of a uniform insertion probability (Barquist *et al.*, 2013). However, there exists some evidence in the literature against this uniform insertion probability assumption (Kimura *et al.*, 2016).

Bio-Tradis is a processing and analysis pipeline by (Barquist *et al.*, 2016) to support the use of TraDIS protocols for identification of essential genes. To make a prediction of gene essentiality, it calculates the insertion index as the number of insertion sites for any gene divided by its length. Based on the assumption that the distribution of the insertion indices across all genes is bimodal with a mode at zero corresponding to essential genes (Langridge *et al.*, 2009), it fits two gamma distributions to the insertion indices corresponding to the two modes of the distribution of the insertion indices. It calculates Log_2_ likelihood ratios (LLR) comparing the likelihoods of the insertion index under the two fitted gamma probability densities. A gene is classified as essential if it has an LLR of less than –2, indicating that it is at least 4 times more likely under the essential gene model than the non-essential one. Similarly, a gene is classified as non-essential if it has an LLR greater than 2. AlbaTraDIS (Page *et al.*, 2020) also builds on Bio-Tradis by adopting a sliding window approach, rather than being dependent on the genome annotation.

TRANSIT (DeJesus *et al.*, 2015) applied the Bayesian method for essentiality analysis. Their Bayesian method uses information on the long consecutive sequences of TA-sites lacking insertions as the variable of interest and has the assumption that insertion gaps of TA-sites occur by chance in non-essential regions, with a geometrical decrement in the probability of a long gap. The Gumbel or Extreme Value distribution was used to model the longest consecutive sequence of TA-sites lacking insertion in a gene. Hence, they identified essential genes by unusual long gaps and using the Bayesian framework, the posterior probability of the longest gap is calculated.

ARTIST (Pritchard *et al.*, 2014) has two pipelines for the analysis of TraDIS data: the Essential Loci Analysis (EL-ARTIST) pipeline and Conditional Essential Loci Analysis (Con-ARTIST) pipeline. The EL-ARTIST pipeline identifies regions that are required for optimal growth under a given condition. It uses a sliding window method to define regions that have low read numbers after normalizing the data for incomplete DNA replication. A hidden Markov model is trained on the results of the sliding window analysis and this refines the prediction of whether each TA site is in a region required or dispensable for growth. ARTIST was developed to analyse TIS datasets generated using mariner-based transposons although the authors comment that it should be adaptable to Tn5 transposons.

Lariviere *et al.* (2020) adopted the approach developed by Goodall *et al.* (2018) which involves fitting known distributions to the distribution of saturation indices. Saturation index was computed by Lariviere *et al.* (2020) as the number of insertions within a coding region divided by the length of the coding region. They applied the Bio-TraDIS package to the distribution of the saturation indices for gene essentiality analysis.

One of the significant limitations in the existing methods is that currently-assumed distributions may not model the insertion variability seen in Tn5-based TraDIS data. The negative binomial or Poisson distributions used to model read counts (Seyednasrollah *et al.*, 2015) may not reflect characteristics of Tn5-based TraDIS data which have greater insertion density and genomic resolution due to the non-preferential insertion of Tn5 transposoons. Another drawback of current methods is the way they handle low-frequency sequencing events (Klein *et al.*, 2012; Le Breton *et al.*, 2015; Yang *et al.*, 2017). The need to develop suitable statistical approaches and computational methods to identify essential bacterial genes using Tn5 transposons is paramount.

Insertion-level-based methods that capitalize on the advantages of mariner transposons dominate the literature of current statistical methods for identification of essential genes. Gene-level methods have not been the focus of so much attention. Nonetheless, some studies have successfully used Tn5 transposons under different growth conditions to classify genes (Barquist *et al.*, 2013; Chao *et al.*, 2016; Christen *et al.*, 2011). This paper presents a novel Bayesian computational method for classifying essential bacterial genes using Tn5-based TraDIS data. It coherently accounts for noise that could lead to spurious findings during statistical analysis. Our gene-level approach uses insertion density as the sole variable in the classification. It avoids the need to arbitrarily set a threshold or to use normalization procedures before analysis like, for example, the trimmed mean method (Zomer *et al.*, 2012).

## 2 Materials and methods

Our approach uses gene-level Tn5 transposon data in the classification procedure. With a large number of transposon insertions, some potential insertion positions could record multiple insertions. Rather than focussing on the total number of insertions per gene we count unique insertions sites (e.g. three insertions in the same position count as one unique insertion site). Given the assumption that transposons inserts randomly within the genome, the number of unique insertion sites for any gene is assumed to increase with gene length so we scale the number of unique insertion sites by gene length. We define the insertion density for a given gene as the number of unique insertion sites divided by the gene length and use insertion density as a classifier of bacterial gene essentiality. We exploit the fact that insertions in essential genes are lethal except at the distal portions, whilst taking into account the fact that what counts as distal in this context will likely vary by gene.

We assume conditional independence of the insertion densities for any two genes given that their essentiality statuses and the values of any relevant parameters. Insertion densities are in the interval [0,1] so we chose to model the probability distribution of insertion density as a beta distribution. For each gene, we derived the posterior probability that the gene is essential using Markov Chain Monte Carlo (MCMC) via both Metropolis–Hastings (MH) and Gibbs sampling as required. MCMC is now a common tool for the analysis of a variety of applied genetic problems (Alenazi *et al.*, 2019; Boggis *et al.*, 2016) We call our model the INSDENS model.

### 2.1 Bayesian model

In this paper, π is used to denote the prior probability distribution; *f* is used to represent the likelihood, full conditional distributions and joint probability distributions; underscore is used to represent a vector or set; the shape and rate parameters are used to parameterize the gamma distribution.

Let $\underline{d}$ represent the set of insertion densities values for all genes. Let *d_i_* represent the insertion density for the *i*th gene. Let *Z_i_* represent a binary indicator variable indicating whether the *i*th gene is essential (*Z_i_* = 1) or not (*Z_i_* = 0). Let *G* be the total number of genes, $\underline{Z}=(Z_{1},Z_{2},Z_{3},\ldots ,Z_{G})$ represent the indicator vector of essentiality for all genes. Let ${\underline{d}}_{E}$ and ${\underline{d}}_{N}$ represent the set of insertion densities for essential and non-essential genes, respectively. Furthermore, *d_Ej_* is the *j*th element of the set *d_E_* with similar meanings for *d_Nj_*. Let *G_E_* be the cardinality of the set ${\underline{d}}_{E}$ and *G_N_* be the cardinality of the set ${\underline{d}}_{N}$.

#### 2.1.1 The likelihood

For essential genes we assume that

(1) $$d_{i}|Z_{i}=1,\alpha_{E},\beta_{E}\;∼\;\text{Beta}(d_{i};\alpha_{E},\beta_{E})$$

and for non-essential genes, we assume that

(2) $$d_{i}|Z_{i}=0,\alpha_{N},\beta_{N}\;∼\;\text{Beta}(d_{i};\alpha_{N},\beta_{N}).$$

Let $\underline{\Theta }=\{\alpha_{E},\beta_{E},\alpha_{N},\beta_{N}\}$. Assuming conditional independence of *d_m_* and *d_n_* given *Z_m_* and *Z_n_* and the parameters, the full likelihood is

(3) $$f(\underline{d}|\underline{Z},\underline{\Theta })=\;\prod_{i=1}^{G}f(d_{i}|Z_{i},\underline{\Theta }).\;$$

#### 2.1.2 The prior distributions

We assume the random variable *Z_i_*, representing the essentiality status for gene *i*, has a Bernoulli probability distribution with parameter *θ*. This, assuming that *Z_i_* is independent of *Z_j_*, a priori, for i≠j implies

(4) $$\pi (\underline{Z}|\theta )=\theta^{G_{E}}{\left(1-\theta \right)}^{G_{N}}.$$

Since 0≤θ≤1 and since θ is uncertain, we place a Beta distribution on θ to capture our prior belief in the uncertainty.

(5) θ∼Beta(0.1,0.9).

This choice of hyperparameters for the theta prior specifies a mean of 0.1 and a monotonically decreasing probability density. Its 10th percentile is approximately $1\times 10^{-10}$ and its 90th percentile is approximately 0.4, so that there is reasonable probability density in the range of values that, a priori, might be anticipated for the proportion of essential genes. For each dataset, we used the observed insertion densities to make a guess at the essentiality of each gene (genes with insertion density below some threshold were assumed to be essential). We calculated the mean and variance of the insertion densities separately for those genes we guessed as essential and those we guessed as non-essential genes. Equating expressions for the mean and variance of the Beta distribution with the sample means and variances of the insertion densities in both groups allows us to obtain estimates of the parameters in $\underline{\Theta }$. This is similar in spirit to an empirical Bayes approach (Spencer *et al.*, 2016) but equating moments is simpler than maximizing marginal likelihoods in this case as it avoids the need for numerical optimization. To allow for uncertainty in the parameter values in $\underline{\Theta }$, we place gamma priors on each of them. We set the rate parameter of each gamma prior to be 1 and the shape parameter equal to the value of the relevant $\underline{\Theta }$ parameter determined my moment matching. This gives a prior with a suitably large variance. For example if, by moment matching, we guessed that a parameter in $\underline{\Theta }$ was 3 our prior for that parameter would be gamma(3,1). The actual priors used are given in Table 1. We also performed a marginal sensitivity analysis for the *Staphylococcus aureus* dataset to determine whether our results were sensitive to the choice of these hyperparameter values.

**Table 1.** Shape parameters of the gamma prior distributions of the parameters in $\underline{\Theta }$

Dataset	Shape parameters of			
	α_*E*_	β_*E*_	α_*N*_	β_*N*_
*E. coli*	2	166	2	17
*S. aureus*	1	182	11	83
*S*. *Typhimurium*	2	178	3	18

#### 2.1.3 Full joint distribution

Combining the full likelihood and prior distributions, the full joint probability distribution for our model is derived as below as:

(6) $$f(\theta ,\underline{\Theta },\underline{Z},\underline{d})=\pi (\theta )\pi (\Theta )\pi (\underline{Z}|\theta )\prod_{i=1}^{G}f(d_{i}|Z_{i},\underline{\Theta }).$$

#### 2.1.4 The conditional distributions

To obtain posterior probabilities of each gene being essential we derived, up to proportionality, the conditional probability densities from which to sample. Using Equations (4) and (5) the conditional distribution for θ is given by

(7) $$f(\theta |\Theta ,\underline{Z},\underline{d})\propto \pi (\theta )\pi (\underline{Z}|\theta )$$

 

(8) $$\propto \theta^{G_{E}-0.9}{\left(1-\theta \right)}^{G_{N}-0.1}$$

which gives

(9) $$\theta |\Theta ,\underline{Z},\underline{d}∼\text{Beta}(0.1+G_{E},0.9+G_{N}).$$

Let $\Theta_{-\psi }$ represent the set $\underline{\Theta }$ excluding the element ψ and let B(.,.) be the usual beta function. Using Equations (1) and (3) and assuming $\alpha_{E}∼\text{Ga}(\phi ,\lambda )$, the conditional distribution for α_*E*_ is given by

(10) $$f(\alpha_{E}|\Theta_{\alpha_{E}},\theta ,\underline{Z},\underline{d})\propto \frac{\alpha_{E}^{f-1}e^{-{\lambda \alpha }_{E}}}{B{\left(\alpha_{E},\beta_{E}\right)}^{G_{E}}}{\left(\prod_{j=1}^{G_{E}}d_{Ej}\right)}^{\alpha_{E}}$$

 

(11) $$\propto \frac{\alpha_{E}^{\phi -1}\;\text{exp}\;\left[-\alpha_{E}\left(\lambda -\sum l\text{og}(d_{Ej})\right)\right]}{B{\left(\alpha_{E},\beta_{E}\right)}^{G_{E}}}$$

where the limits on the summation are the same as those on the product. The conditional distributions of the remaining parameters in $\underline{\Theta }$ can be obtained in a similar manner and are detailed in Supplementary File S1. The full conditional for *Z_i_* is a $\text{Bernoulli}\left(\frac{p_{E}}{p_{E}+p_{N}}\right)$ probability distribution where

(12) $$p_{E}=\theta \prod_{j=1}^{G_{E}}f(d_{Ej}|Z_{j}=1,\Theta )$$

 

(13) $$p_{N}=(1-\theta )\prod_{j=1}^{G_{N}}f(d_{Nj}|Z_{j}=0,\Theta )$$

The posterior probability of gene *i* being essential is calculated as the proportion of MCMC iterations (ignoring burn-in) where it is classified as being essential.

#### 2.1.5 Sampling from the posterior distributions

We used the a random-walk MHs algorithm to sample from the posterior distribution of the parameters in $\underline{\Theta }$ and Gibbs sampling to sample from the posterior distributions of $\underline{Z}$ and θ. We update each element of $\underline{Z}$ sequentially. After sampling *Z_i_* both *p_E_* and *p_N_* are recalculated to take into account which genes are currently considered to be essential.

We use the following procedure when using the MH algorithm to update each of the parameters in $\underline{\Theta }$: Let Θ_*ic*_ and Θ_*ip*_ be the current and proposed value of the *i*th parameter in $\underline{\Theta }$, respectively. The proposed value is obtained by drawing a random deviate *h* from a *N*(0, 1) distribution and then calculating $\Theta_{ip}=\Theta_{ic}\;\text{exp}(\sigma^{2}\times h)$ where $\sigma^{2}$ is tuned to give an appropriate acceptance rate of around 30%. Since the proposal density is symmetric in Θ_*ic*_ and Θ_*ip*_, the MH ratio is

(14) $$\gamma (\Theta_{ip},\Theta_{ic})=\frac{f(\Theta_{ip}|\Theta_{-i},\theta ,\underline{Z},\underline{d})}{f(\Theta_{ic}|\Theta_{-i},\theta ,\underline{Z},\underline{d})}$$

We sample a realization *u* of a uniform[0, 1] distribution and accept the proposed value Θ_*ip*_ with probability $\text{min}(1,\gamma (\Theta_{ip},\Theta_{ic}))$. After some experimentation, we set the initial value of $\sigma^{2}$ of the MHs sampling procedure for the parameters α_*E*_, β_*E*_, α_*N*_ and β_*N*_ as 0.25, 0.30, 0.04 and 0.05, respectively and manually modified each value to get an acceptable acceptance rate.

We selected multiple sets of initial values for each chain to check for convergence. We performed a quantitative diagnostic check for convergence by calculating the potential scale reduction factor values for all the parameters in $\underline{\Theta }$. All values were sufficiently close to 1 to indicate convergence.

We specified the burn-in to be 2000 iterations. This is highly conservative, but we wanted to be sure that the retained values were from the posterior distribution in all analyses. Some of the parameters showed moderate autocorrelation. Current practice is not to thin the samples to reduce the autocorrelation but instead to run the chain for longer. We ran the MCMC sampler for a 22 000 so that after discarding burn-in we were left with 20 000 iterations to base the posterior probabilities on.

We initialized the probability of a gene being essential as θ=0.1 corresponding to a 10% chance that any given gene is essential. We initialized the values of the parameters α_*E*_, β_*E*_, α_*N*_ and β_*N*_ to be the values specified in Table 1. We initialized the essentiality assignment, *Z_i_*, to be 1 if the insertion density for that gene was below 0.025 and 0 otherwise.

#### 2.1.6 Real datasets

We apply our INSDENS model to three TraDIS datasets: *Escherichia coli*; *Salmonella* Typhimurium and *S. aureus*. The *E. coli* K-12 BW25113 data (Goodall *et al.*, 2018) were sourced from the European Nucleotide Archive under accession number ERR2249109. The *E. coli* K-12 BW25113 data has a total number of 448 854 insertions. The list of *E. coli* K-12 genes likely to be essential was obtained from Baba *et al.* (2006), and the likely non-essential genes were genes for which a deletion mutant was successfully generated in that study.

The *S*. Typhimurium data used in this study was based on the work of Barquist *et al.* (2013) and was sourced from the European Nucleotide Archive under accession numbers ERR009073 and ERR009074. The *S*. *Typhimurium* data has a total of 639969 distinct transposon insertions mapped to its genome. For the *S*. *Typhimurium* dataset, the genes we listed as essential were homologues of the genes from *S. Typhimurium* ST14028 which Porwollik *et al.* (2014) attempted to knock out using two different approaches and were unsuccessful, and also homologues of *E. coli* K-12 genes which could not be knocked out by Baba *et al.* (2006). The genes listed as non-essential were homologues of genes which had mapped knockout mutations by both approaches in *S. Typhimurium* ST14028 (Porwollik *et al.*, 2014), with the exclusion of six genes which were homologues of *E. coli* K-12 genes defined as essential by Baba *et al.* (2006).

The *S. aureus* data (Christiansen *et al.*, 2014) were sourced from the database under accession numbers SRR105406 to SRR1056422. The genes designated as essential were homologues of genes which were identified as essential both in Fey *et al.* (2013) and Chaudhuri *et al.* (2009). The non-essential genes were defined as genes which had mutations in the Fey *et al.* (2013) study.

All datasets were re-analysed using a common pipeline. Transposon tag sequences (where present) were removed using Cutadapt (Martin, 2011). The tag-free reads were mapped to the reference genome using BWA mem (Li, 2013). The mapped position of the 5’ end of each read was determined using bedtools bamtobed (Quinlan and Hall, 2010), and used to infer the position of transposon insertions. The UNIX tool uniq was used to count the number of reads associated with each unique insertion site.

#### 2.1.7 Synthetic datasets

We simulated four different datasets to mimic four scenarios of interest. Each dataset contained 4000 genes of which 200 were essential. The datasets were generated using properties of the *E. coli* TraDIS dataset. In the *E. coli* dataset, we observed that the variance in the number of insertions by gene increases with gene length (Supplementary File S2). To mimic this behaviour, we implemented the following procedure: initialize the gene-specific insertion probability to be the gene length divided by the total gene length across all genes; generate a multiplicative factor for each gene from a beta(5, 4) distribution; randomly multiply or divide the gene-specific insertion probability by this multiplicative factor. We determined the number of insertions for each gene by randomly sampling genes according to this adjusted insertion probability. Insertions in non-essential genes were inserted at random anywhere in the gene. For essential genes, the insertion probability decreased with distance from the gene ends. Specifically we sample values from an exponential distribution with rate parameter λ. These sampled values represent the distance from the gene ends relative to the gene length. We considered λ  =  50 and λ  =  10. When λ  =  50 we expect more than 99% of insertions to be within 10% of the gene ends. When λ  =  10 this value drops to 63%. We also incorporated noise into our simulations by allowing spurious insertions to insert anywhere in both essential and non-essential genes.

We label our four scenarios as HH, HL, LH and LL. The first letter specifies whether the total number of insertions across all genes is high (H) or low (L). High corresponds to 700 000 insertions and low to 20 000 insertions. The second letter specifies whether the exponential rate parameters is high (H) or low (L). High corresponds to λ  =  50, low to λ  =  10. In all scenarios, we specified the number of spurious reads to be approximately 5% of the number of true insertions. Table 2 shows the different combinations used. The total number of unique insertions for the *E. coli*, *S. aureus* and *S*. *Typhimurium* datasets are approximately 347 133 and 353 000, respectively.

**Table 2.** Numbers of true insertions, spurious insertions and exponential rate parameter, λ, for the four simulated data scenarios

Dataset	Number of Insertions	Number of spurious insertions	λ
HH	700 000	30 000	50
HL	700 000	30 000	10
LH	20 000	1000	50
LL	20 000	1000	10

#### 2.1.8 Comparative study

We used receiver operating characteristic (ROC) curves to compare the classification performance of INSDENS in both our real and simulated datasets. When comparing the performance of INSDENS versus Bio-Tradis for our real datasets we compared the true positive rates (TPRs) and false positive rates (FPRs). Rather than having to place an arbitrary threshold on the posterior probability of essentiality, we used Bayesian decision theory which allows us to assign costs to making a decision (gene is essential or non-essential), given the truth (gene is essential or non-essential). We assume that there is no cost in making the correct decision. There are, therefore, two costs to assign: false discovery cost (*C_A_*) when INSDENS incorrectly identifies a gene as essential when it is actually non-essential and the false non-discovery cost (*C_B_*) when INSDENS identifies a gene as non-essential when it is actually essential (Walters *et al.*, 2021). Bayesian decision theory states that gene *i* is declared to be essential if the posterior cost of declaring it to be essential is less than the posterior cost of declaring it be non-essential. If *p_i_* is the posterior probability of gene *i* being essential then this inequality becomes $(1-p_{i})C_{A}<p_{i}C_{B}$ which leads to the following classification rule: gene *i* is classified as essential if

(15) $$p_{i}>R=\frac{C_{A}}{C_{A}+C_{B}}=\frac{1}{1+Q}.$$

where $Q=C_{B}/C_{A}$ is a ratio of costs.

## 3 Results

### 3.1 Analysis of real datasets

**Fig. 1.**
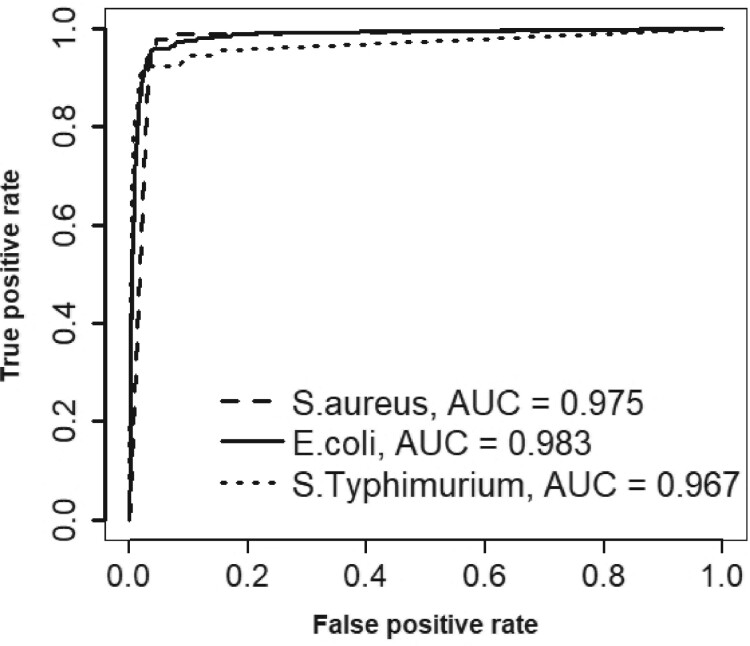
ROC curves and AUCs (in the legend) for the *E. coli*, *S*. *Typhimurium* and *S. aureus* datasets using the posterior probability of gene essentiality as the classifier

The ROC curves for the INSDENS model applied to the *E. coli*, *S. aureus* and *S*. *Typhimurium* datasets are shown in Figure 1. The area under the curve (AUC) values are 0.975, 0.983 and 0.967 for the *S. aureus*, *E. coli* and *S*. *Typhimurium* datasets, respectively. We investigated the effect of changing one hyperparameter at a time (marginal sensitivity analysis) for the *S. aureus* dataset to determine the sensitivity of the results to the hyperparameter value. We took each of the four values for *S. aureus* in Table 1 one at a time and multiplied it by 2 and then by 0.5, keeping all the other values fixed. This require eight runs of the MCMC routine. The sensitivity analysis conducted showed that the AUCs are not very sensitive to changes in the hyperparameters with all 8 AUCs between 0.973 and 0.977 inclusive.

### 3.2 Analysis of synthetic datasets

**Fig. 2.**
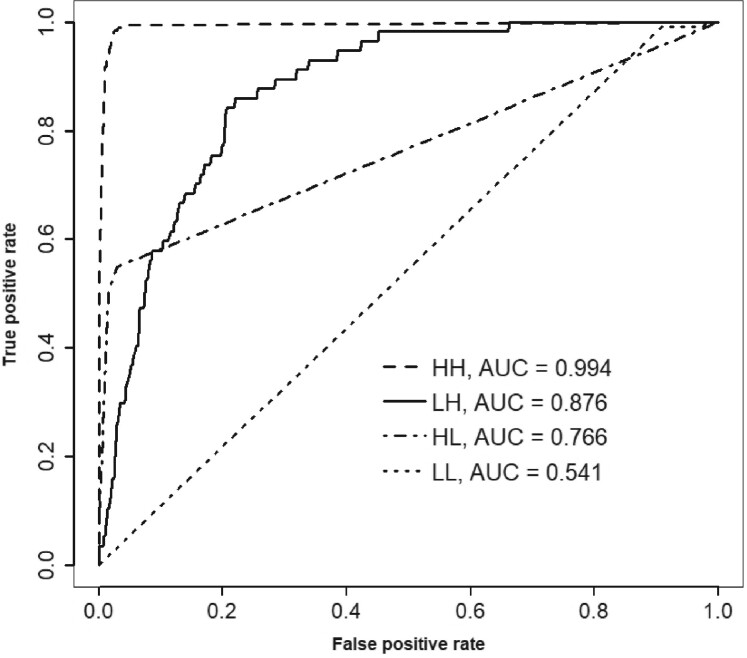
The ROC curves for the simulated HH, LH, HL and LL datasets. The AUCs are given in the legend. The classification statistic is the posterior probability of gene essentiality (**a**) *E. coli*, (**b**) *S*. *Typhimurium* and (**c**) *S. aureus*

The ROC curves when our model is applied to the synthetic datasets are shown in Figure 2. The AUCs are 0.994, 0.876, 0.766 and 0.541 for HH, LH, HL and LL, respectively. The AUC values in the four simulated scenarios are affected by both the number of insertions (AUC increases with the number of insertions) and how far from the gene ends insertions are tolerated in essential genes (AUC increases as this tolerance decreases). Our simulation software could be used as a tool to estimate the likely classification performance using our MCMC procedure based on simple dataset-specific characteristics.

### 3.3 Comparison with bio-tradis

**Fig. 3.**
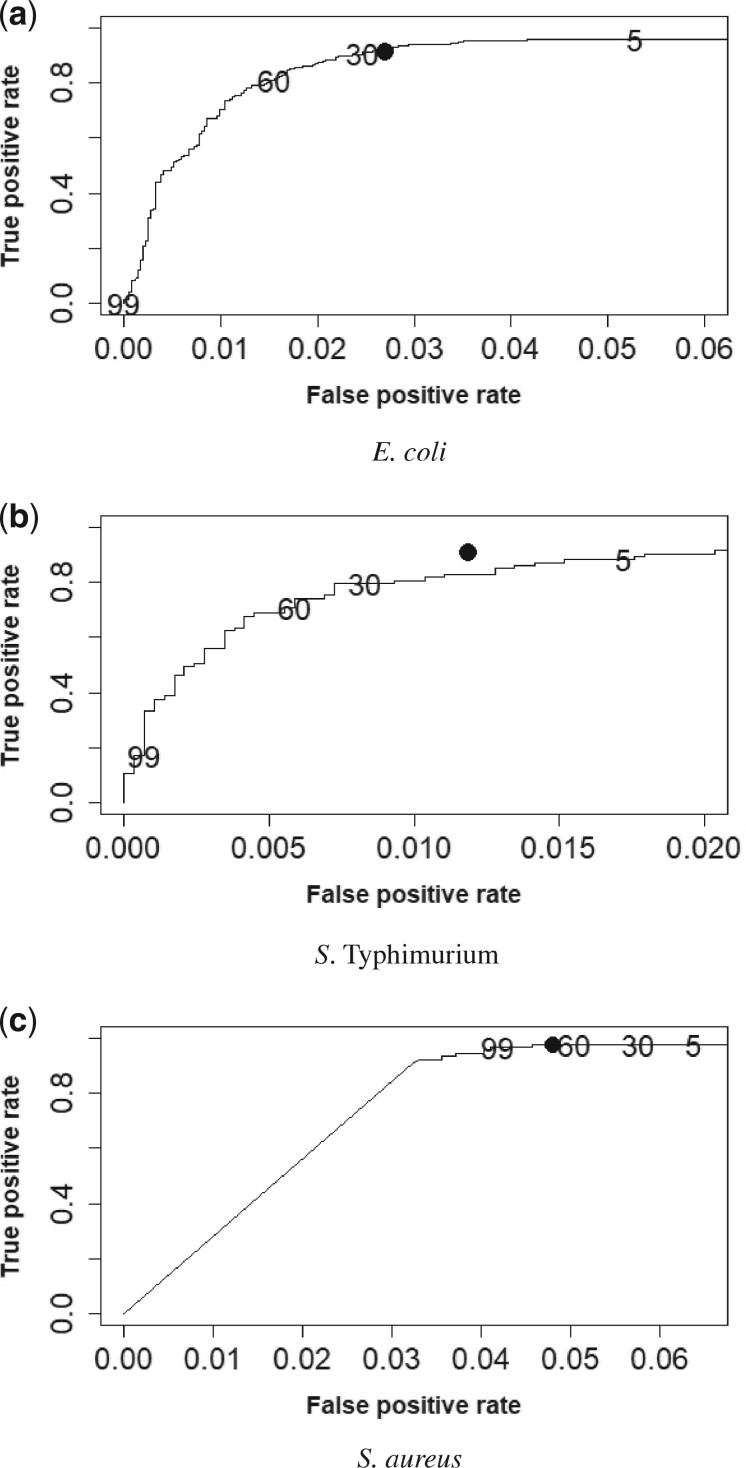
True and false positive rates for Bio-Tradis and INSDENS for four different values of $R=C_{A}/(C_{A}+C_{B})$. The point in ROC space is labelled with its numerical *R* value for INSDENS or with a solid circle for Bio-Tradis. For convenience, the *R* value shown is 100 times the actual value

When calculating the FPRs and TPRs for INSDENS we used four values of $C_{A}(5,30,60,99)$ and $C_{B}(95,70,40,1)$ in the Bayesian decision theory approach. These require the posterior probability to exceed R=0.05,0.30,0.60 and 0.99, respectively, for the gene to be declared essential. The FPRs and TPRs for INSDENS and Bio-Tradis when both methods were applied to the *E. coli*, *S*. *Typhimurium* and *S. aureus* datasets are shown in Figure 3. Each plot shows four points for INSDENS, one for each value of $R=C_{A}/(C_{A}+C_{B})$, and a single point for Bio-Tradis. We observe from Figure 3 that the FPRs and TPRs of the Bio-Tradis analyses are on or close to the ROC curves for each of the three datasets. We also see that our method affords considerable flexibility in the TPRs and FPRs depending on the costs *C_A_* and *C_B_*.

## 4 Discussion

Our new method for classifying bacterial genes using TraDIS data avoids the need to arbitrarily set thresholds or use normalization procedures before analysis. It also avoids giving a hard classification as, for example, Bio-Tradis does. This gives the user more flexibility in determining which set of genes might be essential. Using our approach it is possible to select fewer genes by decreasing *Q*. This would lead to a reduction in the both the TPR and FPR. Whilst Bio-Tradis performs well, it offers no control over the number of genes classified as essential.

Choosing the costs in a Bayesian decision theory approach is not routinely undertaken and some users may feel unable to confidently attach such costs. In this case, we suggest conducting a sensitivity analysis to see how the selected essential gene set changes as the costs of making incorrect decisions vary. Conceptually, the simplest way to do this is to vary the value of *Q*, which measures how much more costly it is to misclassify an essential gene than to misclassify a non-essential gene. A user might choose a range of values of *Q* say $Q_{\text{min}}<Q<Q_{\text{max}}$ and monitor how the set of genes declared essential varies for values of *Q* in this range. Alternatively users can put their own threshold on the posterior probability or ranks genes using it.

Running 10 000 MCMC iterations on a desktop PC with a single 1.8 GHz processor with 8 GB of RAM took 100, 80 and 30 s for the *E. coli*, *S*. *Typhimurium* and *S. aureus* datasets, respectively. It may be possible to reduce the run-time further using importance sampling or sampling importance re-sampling, provided a suitable choice of importance distribution can be found.

There is further information that could improve the classification of essential genes. Our current approach is potentially susceptible to noise since we do not leverage read count information in our model. Doing so would allow us to better distinguish true reads from spurious reads and might lead to further classification performance with relatively little computationally cost.

## Funding

This work was supported by the Nigerian Government through the NEEDS Assessment Scholarship/Fellowship [FUT/DVC(Acad.)/GEN 101/1/18].

*Conflict of Interest*: none declared.

## Supplementary Material

btab508_Supplementary_Data
