# Corrigendum to: Probabilistic identification of bacterial essential genes via insertion density using TraDIS data with Tn5 libraries

**DOI:** 10.1093/bioinformatics/btab610

**Published:** 2021-09-14

**Authors:** Valentine U Nlebedim, Roy R Chaudhuri, Kevin Walters

*Bioinformatics* (2021) doi: 10.1093/bioinformatics/btab508

In the published version, co-author Kevin Walters’ affiliation is incorrect. This has now been corrected online.

