# Supplementary material for: Probabilistic identification of bacterial essential genes via insertion density using TraDIS data with Tn5 libraries
Source: Bioinformatics. 2021 Jul 13;37(23):4343–9. doi: 10.1093/bioinformatics/btab508 (PMC8652038; doi:10.1093/bioinformatics/btab508)
Supplement: btab508_Supplementary_Data [file btab508_supplementary_data.zip › Supplementary file 1.pdf]

Denote the gamma prior shape and rate hyperparameters for each parameter in $\underline{\Theta}$ as ϕ and λ. The full conditionals for the parameters in $\underline{\Theta}$ are given in Table 1. Note that the actual values of ϕ for each parameter in $\underline{\Theta}$ are given in Table 1 in the main text. The value of the rate prior hyperparameter is set to $\lambda = 1$ in all cases.

Parameter	Conditional Distribution
α_E	$\alpha_E^{\phi-1} exp\left\{-\alpha_E\left[\lambda - \sum \log(d_{Ej})\right]\right\}/B(\alpha_E, \beta_E)^{G_E}$
β_E	$\beta_E^{\phi-1} exp\left\{-\beta_E\left[\lambda G_E \sum \log(1-d_{Ej})\right]\right\}/B(\alpha_E, \beta_E)^{G_E}$
α_N	$\alpha_N^{\phi-1} exp\left\{-\alpha_N\left[\lambda - \sum \log(d_{Nj})\right]\right\}/B(\alpha_N, \beta_N)^{G_E}$
β_N	$\beta_N^{\phi-1} exp\left\{-\beta_N\left[\lambda G_E \sum \log(1-d_{Nj})\right]\right\}/B(\alpha_N, \beta_N)^{G_E}$

Table 1. Conditional distributions of the parameters in $\underline{\Theta}$
