# Supplementary material for: Probabilistic identification of bacterial essential genes via insertion density using TraDIS data with Tn5 libraries
Source: Bioinformatics. 2021 Jul 13;37(23):4343–9. doi: 10.1093/bioinformatics/btab508 (PMC8652038; doi:10.1093/bioinformatics/btab508)
Supplement: btab508_Supplementary_Data [file btab508_supplementary_data.zip › Supplementary file 4.pdf]

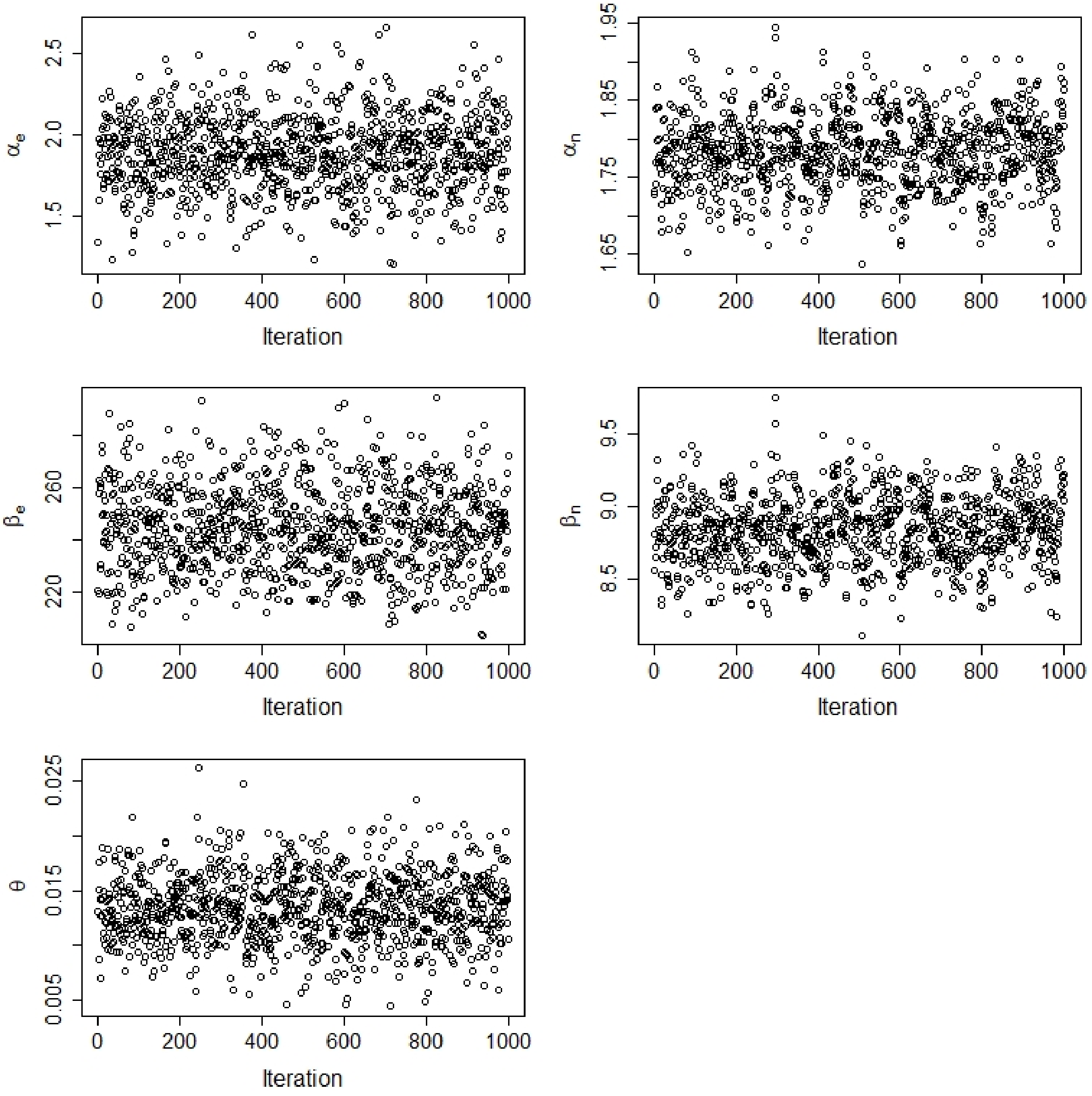

Figure 1. Trace plots for the parameters $\alpha_E, \alpha_N, \beta_E, \beta_N$ and θ for the HH simulated data MCMC analysis whose ROC curve is shown in Figure 3 in the main text.

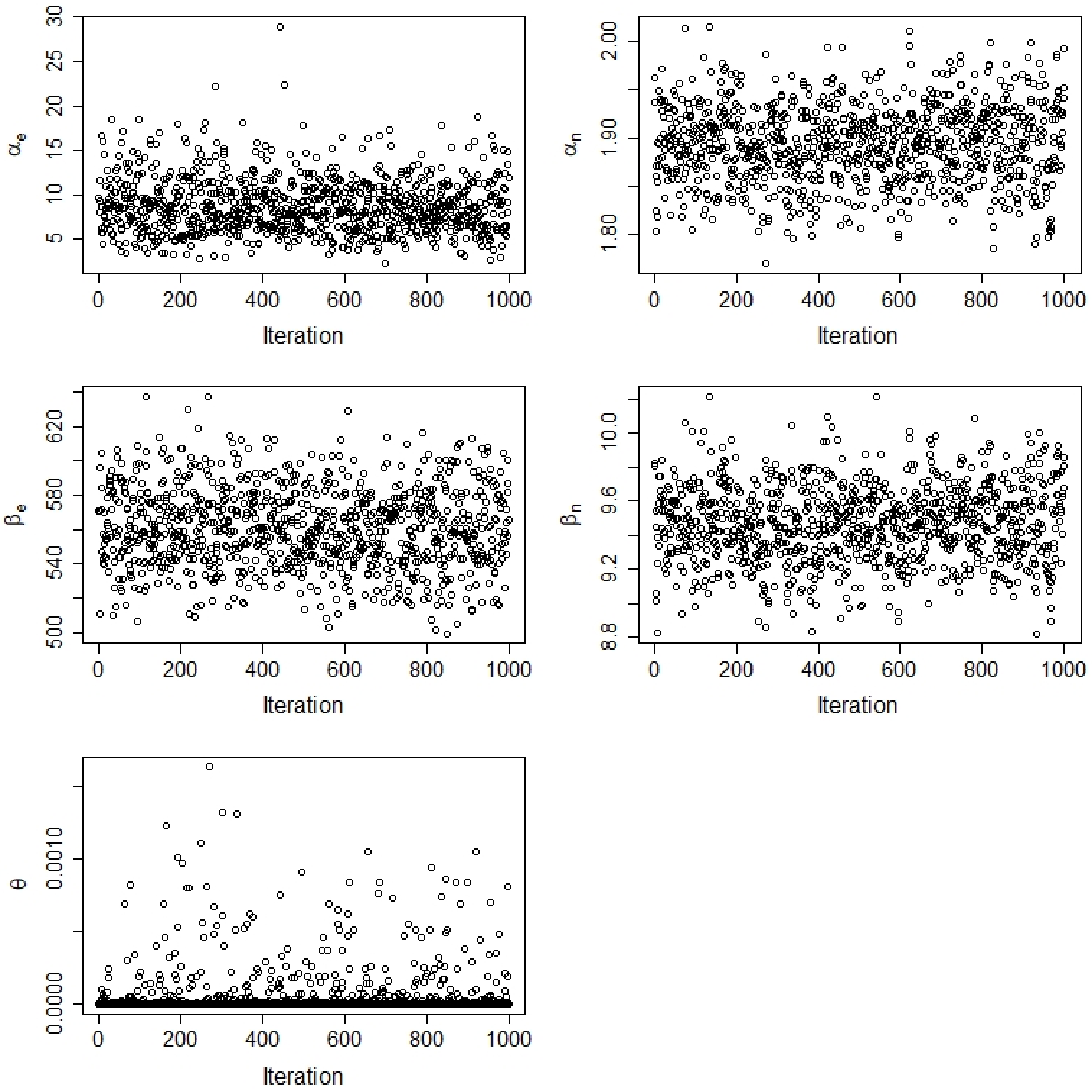

Figure 2. Trace plots for the parameters $\alpha_E, \alpha_N, \beta_E, \beta_N$ and θ for the HL simulated data MCMC analysis whose ROC curve is shown in Figure 3 in the main text.

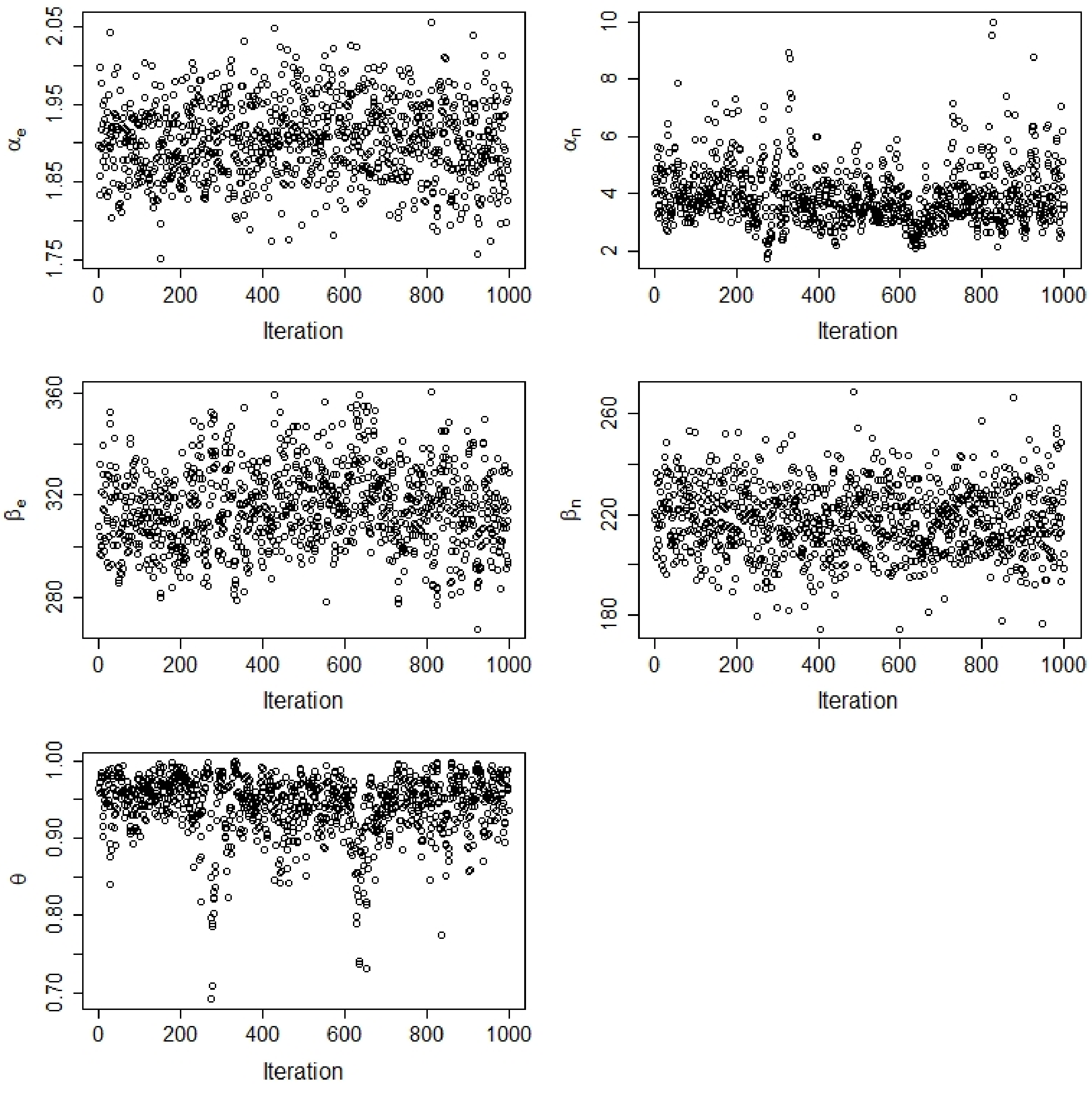

Figure 3. Trace plots for the parameters $\alpha_E, \alpha_N, \beta_E, \beta_N$ and θ for the LH simulated data MCMC analysis whose ROC curve is shown in Figure 3 in the main text.

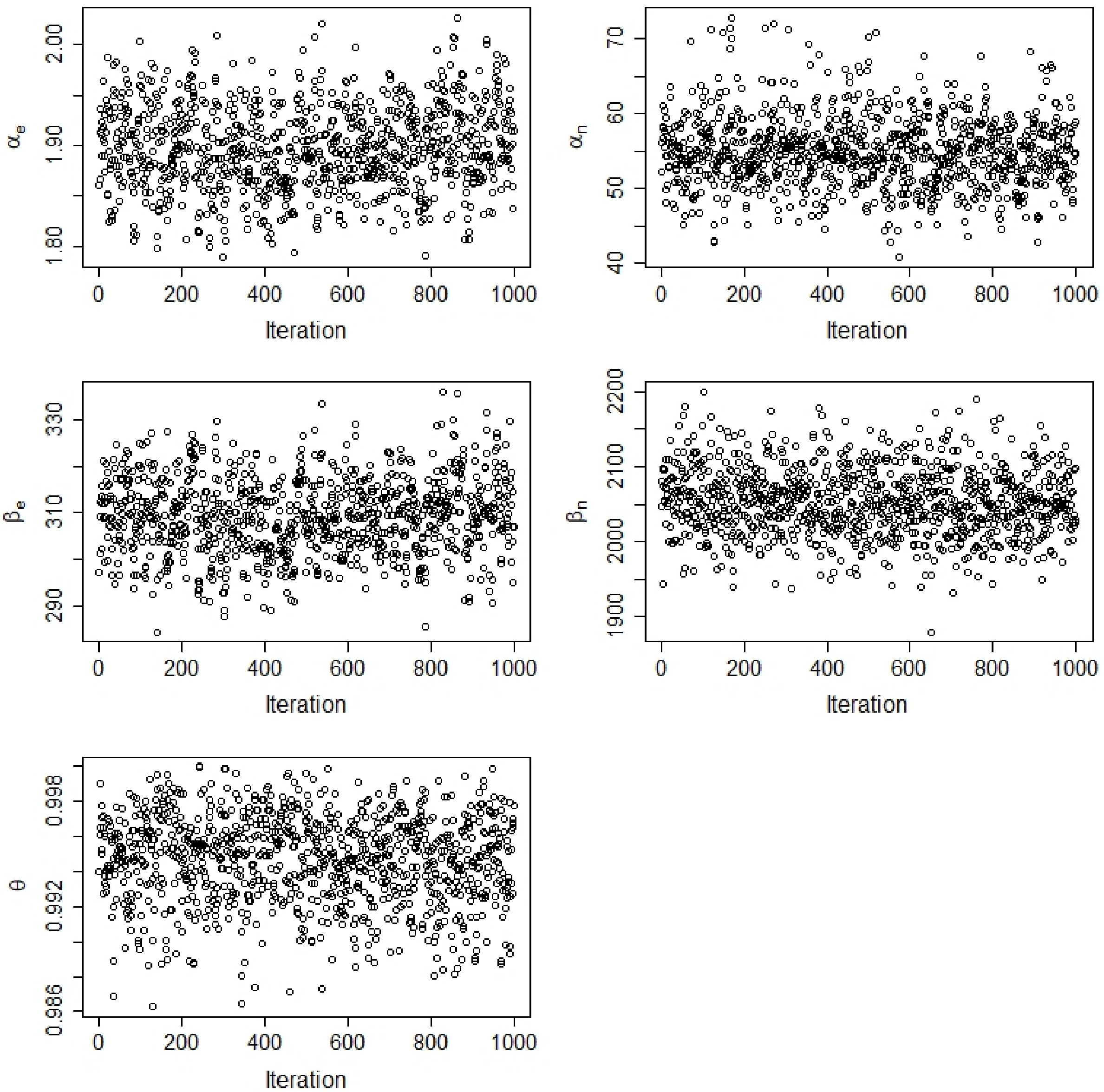

Figure 4. Trace plots for the parameters $\alpha_E, \alpha_N, \beta_E, \beta_N$ and θ for the LL simulated data MCMC analysis whose ROC curve is shown in Figure 3 in the main text.
