# Supplementary figures and images for: Probabilistic identification of bacterial essential genes via insertion density using TraDIS data with Tn5 libraries
Source: Bioinformatics. 2021 Jul 13;37(23):4343–9. doi: 10.1093/bioinformatics/btab508 (PMC8652038; doi:10.1093/bioinformatics/btab508)

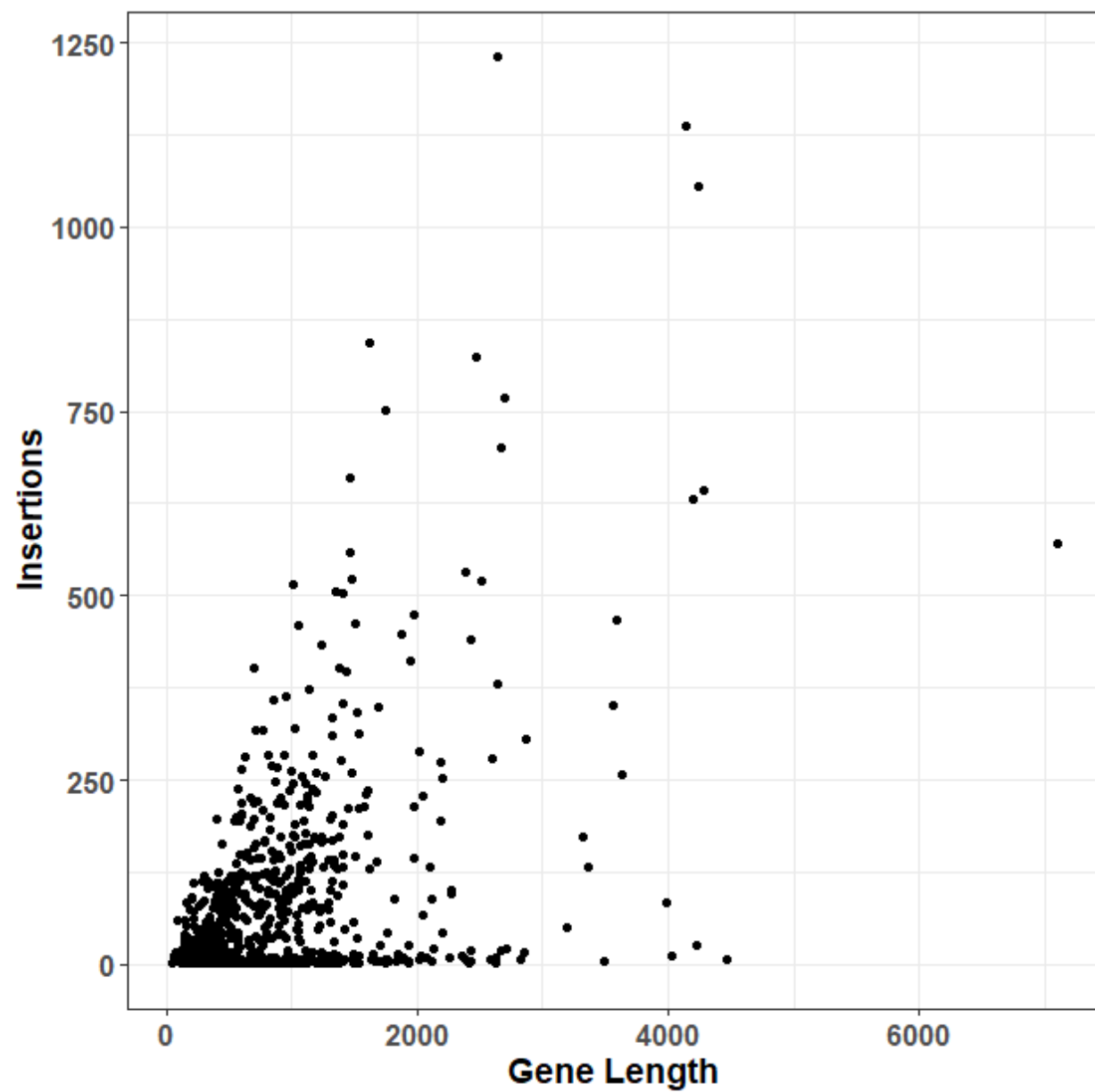

**Figure 1.** Gene-length versus total number of insertions by gene for non-essential *E. coli* genes.

Supplement: btab508_Supplementary_Data [file btab508_supplementary_data.zip › Supplementary file 2.pdf]
